# Blood Lipid Levels and the Severity of Guillain–Barré Syndrome: A Single-Center Retrospective Cohort Study

**DOI:** 10.1155/nri/1098949

**Published:** 2025-08-07

**Authors:** Yangrongzhuo Huang, Lina Feng, Yuhan Li, Hailing Zhou, Linglong Meng, Xuening Li, Juan Tang

**Affiliations:** ^1^Department of Neurology, The First Affiliated Hospital of Shihezi University, Shihezi, China; ^2^Department of Preventive Medicine, School of Medicine, Shihezi University, Shihezi, China

**Keywords:** apolipoprotein, cranial nerve involvement, disease severity, Guillain–Barré syndrome, lipid metabolism, low-density lipoprotein (LDL)

## Abstract

**Objective:** To investigate the association between lipid profiles and disease severity/cranial nerve involvement in Guillain–Barré syndrome (GBS), providing evidence for early clinical intervention.

**Methods:** This retrospective study enrolled 182 GBS patients (148 males and 34 females) admitted to the First Affiliated Hospital of Shihezi University from December 2019 to April 2024. Patients were stratified into mild (Hughes Functional Disability Scale [HFDS] 1–3) and severe (HFDS 4–6) groups. Multivariate logistic regression (adjusted for age, sex, and antecedent infections) was used to analyze independent associations of low-density lipoprotein cholesterol (LDL-C) and apolipoprotein A (ApoA) with disease severity and cranial nerve involvement. ROC curve analysis determined predictive thresholds.

**Results:** Disease severity: each 1 mmol/L increase in LDL elevated severe disease risk by 2.5-fold (OR = 2.503, *p*=0.009) and each 0.1 g/L decrease in ApoA reduced severe disease risk by 99.6% (OR = 0.004, *p* < 0.001). Cranial nerve involvement: LDL ≥ 2.355 mmol/L significantly increased cranial nerve involvement risk (OR = 1.925, *p*=0.018). Predictive thresholds: LDL ≥ 2.215 mmol/L optimally predicted severe disease and ApoA ≤ 1.071 g/L indicated higher probability of mild disease.

**Conclusion:** Elevated LDL and reduced ApoA are independent risk factors for GBS progression and cranial nerve involvement. Combined detection may aid early identification of high-risk patients. Dyslipidemia likely exacerbates GBS pathology through neuroinflammatory mechanisms, suggesting targeted lipid regulation as a potential therapeutic strategy.

## 1. Introduction

Guillain–Barré Syndrome (GBS) is an immune-mediated disorder of the peripheral nervous system characterized by myelin sheath or axonal damage. Its classic presentation includes rapidly progressive symmetric limb paralysis with or without cranial nerve impairment [1], accompanied by diminished or absent deep tendon reflexes. The annual incidence is approximately 1-2 cases per 100,000 population [2]. Although the exact pathogenesis remains incompletely understood, the prevailing hypothesis suggests that molecular mimicry triggered by pathogen infections (e.g., viruses or bacteria) plays a pivotal role. Specifically, structural homology between pathogen surface antigenic epitopes and peripheral nerve myelin-specific antigens initiates cross-reactive immune responses. This process activates autoreactive T cells and induces specific autoantibody production, which synergistically attack peripheral or central neural structures. Dysregulated complement activation mediates demyelination or axonal degeneration, forming the hallmark pathological features of GBS. The prognosis remains suboptimal, with approximately 20% of the patients exhibiting persistent severe disability 6 months postonset [1], and life-threatening complications may occur. Early assessment of disease severity through serological biomarkers and timely interventions are, therefore, critical for therapeutic management and prognostic improvement.

Blood lipids encompass cholesterol and triglycerides (TGs). Cholesterol is functionally categorized into low-density lipoprotein cholesterol (LDL-C): transported via apolipoprotein B (ApoB) to deliver cholesterol from the liver to peripheral tissues, high-density lipoprotein cholesterol (HDL-C): mediated by apolipoprotein A (ApoA) to reverse transport excess cholesterol from peripheral tissues to the liver for clearance, and very-low-density lipoprotein cholesterol (VLDL-C): primarily transports TGs and is metabolized into LDL-C in circulation. The myelin sheath contains 70%–80% lipids, making dysregulated blood lipid levels or impaired myelin lipid metabolism a potential contributor to neurological disorders such as multiple sclerosis [3]. Emerging evidence suggests that lipid metabolism disturbances—manifested as abnormal LDL-C or HDL-C levels—may serve as biomarkers for assessing GBS severity and prognosis [4]. A study identified significant correlations between aberrant LDL/HDL levels and GBS clinical progression [5]. However, the mechanistic links between lipid metabolism and GBS pathophysiology remain underexplored. This study investigates the association of lipid profiles (including apolipoproteins) with GBS disease severity and cranial nerve involvement, aiming to provide a theoretical foundation for early prevention and targeted clinical interventions.

## 2. Subjects and Methods

### 2.1. Subjects

This retrospective study was approved by the Ethics Committee of the First Affiliated Hospital of Shihezi University (approval no.: KJ2024-191-01). The cohort comprised 182 hospitalized patients diagnosed with GBS at the same institution between December 2019 and April 2024, who met predefined inclusion and exclusion criteria. Written informed consent was obtained from all participants.

Inclusion criteria: diagnosis of GBS or its variants according to international criteria [6], age < 90 years.

#### 2.1.1. Exclusion Criteria

1. Concomitant neurological disorders affecting cerebral function (e.g., subarachnoid hemorrhage and encephalitis);
2. History of neurological sequelae (e.g., cerebral infarction and Alzheimer's disease) interfering with accurate GBS severity assessment;
3. Prior use of lipid-lowering agents or medications influencing lipid profiles;
4. Comorbid hepatic or renal diseases;
5. History of exposure to or use of specific medications;
6. Hysterical paralysis secondary to psychiatric disorders.

### 2.2. Methods

#### 2.2.1. Patient Information and Data Collection

This study comprehensively documented baseline characteristics of enrolled patients (including age, gender, and season of onset), medical history (covering current illness, past chronic/autoimmune diseases, medication history within 3 months preonset, and infections within 4 weeks preonset), clinical manifestations (e.g., initial symptom types, motor dysfunction, sensory abnormalities, cranial nerve involvement, and autonomic disturbances), and serological profiles (lipid metabolism markers and so on). Apolipoprotein-related ratios were defined as follows: Apo A/B ratio = ApoA (g/L)/ApoB (g/L), LDL-ApoB ratio = LDL-C (mmol/L)/ApoB (g/L), and HDL-ApoA ratio = HDL-C (mmol/L)/apolipoprotein A1 (ApoA1) (g/L).

#### 2.2.2. Disease Severity Assessment

Neurological disability severity was evaluated using the Hughes Functional Grading Scale (HFGS) [7] at peak disease severity (defined as the nadir of muscle strength or requirement for mechanical ventilation). Based on HFGS scores, patients were stratified into two groups: mild group (scores 1–3) and severe group (scores 4–6).

#### 2.2.3. Serological Indicators

Fasting venous blood samples were collected from patients on the morning following hospital admission. The following serological parameters were analyzed: lipid profile: total cholesterol (TC), TGs, LDL, HDL, ApoA, ApoB, lipoprotein a (Lp(a)), and liver function markers: total bilirubin (TBil), direct bilirubin (DBil), and other relevant hepatic indicators.

### 2.3. Statistical Analysis

For quantitative data, this study employed the Kolmogorov–Smirnov test to assess whether the data conformed to a normal distribution. Data that followed a normal distribution were expressed as the mean ± standard deviation (Mean ± SD), and independent samples *t*-tests were used to compare differences between groups. Data that did not follow a normal distribution were expressed as median and interquartile range *M* (*Q*1–*Q*3), and nonparametric Mann–Whitney *U* tests were utilized for analysis. Categorical variables were presented as *n* (%), and Chi-square tests or Fisher's exact tests were applied for analysis. A logistic regression model was used to explore the relationship between lipid levels and disease severity as well as cranial nerve involvement. Significant indicators were plotted on receiver operating characteristic (ROC) curves to determine cutoff values. All statistical analyses and graph generation were performed using SPSS and Origin software, with a *p* value < 0.05 considered statistically significant.

## 3. Result

### 3.1. Correlation Between Lipid Levels and Severity of Disease

#### 3.1.1. Comparison of General Information of Patients With Severe and Mild Illnesses

After applying inclusion and exclusion criteria, 182 GBS patients were enrolled, including 70 mild cases (38.46%) and 112 severe cases (61.54%). Statistically significant differences (*p* < 0.05) were observed between groups in age, cranial nerve involvement, gamma-glutamyl transferase (GGT), alkaline phosphatase, TC, LDL, HDL, ApoA, ApoB, sensory impairment, motor impairment, diabetes, and hypertension. No statistically significant differences (*p* > 0.05) were found in total protein (g/L), alanine aminotransferase (ALT), aspartate aminotransferase (AST), TBil, DBil, indirect bilirubin, creatinine, uric acid, TGs, ApoB, Lp(a), or sex distribution (Table 1). Among them, mild cases included 20 patients (28.6%) involving a single cranial nerve and 2 patients (2.9%) involving multiple groups of cranial nerves. Severe cases included 24 patients (21.4%) involving a single cranial nerve and 66 patients (58.9%) involving multiple groups of cranial nerves.

#### 3.1.2. Logistic Regression Analyses for Severely and Mildly Ill Patients

Univariate logistic regression analysis incorporating hypertension, diabetes, lipid profiles, apolipoprotein levels, and liver function parameters demonstrated statistically significant differences (*p* < 0.05) between severe and mild groups for diabetes, hypertension, LDL, ApoA, and ApoB. No significant differences (*p* > 0.05) were observed in other evaluated parameters (Table 2).

Variables demonstrating significance in univariate analysis—hypertension, diabetes, LDL, ApoA, and ApoA/B—were included in a multivariate logistic regression model. The results revealed that LDL (OR = 2.503, 95% CI: 1.259–4.977, and *p* < 0.05) and ApoA (OR = 0.004, 95% CI: 0.001–0.073, and *p* < 0.01) independently correlated with disease severity. Specifically, LDL showed a positive association with worsening clinical severity; ApoA exhibited a negative association with disease progression. Other variables (e.g., hypertension, diabetes, and ApoA/B) lost statistical significance in the adjusted model (*p* > 0.05) (Table 3).

#### 3.1.3. Predictive Efficacy of Lipid Levels on Disease Severity

**Figure 1:**
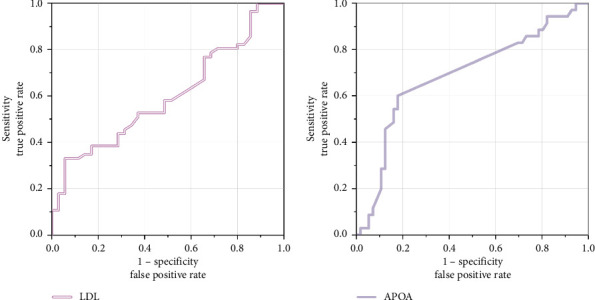
ROC curves for LDL and ApoA in predicting disease severity.

The diagnostic efficacy of LDL and ApoA was analyzed using ROC curves (Figure 1). The results showed that when predicting severe disease, the AUC for LDL was 0.599 (95% CI: 0.517, 0.681), *p*=0.024 (< 0.05), with an optimal cutoff value of 2.215 mmol/L, sensitivity of 82.1%, and specificity of 17.1%, suggesting that LDL levels ≥ 2.215 mmol/L may indicate more severe disease. When predicting mild disease, the AUC for ApoA was 0.690 (95% CI: 0.607, 0.772), *p* < 0.001, with an optimal cutoff value of 1.071 mg/dL, sensitivity of 60.0%, and specificity of 82.1%, indicating that Apo A levels ≤ 1.071 mg/dL may suggest milder disease.

This figure presents the ROC curves for LDL and ApoA in predicting disease severity. The area under the curve (AUC) reflects the predictive performance of each biomarker, with an AUC closer to 1 indicating better predictive efficacy.

### 3.2. Correlation Between Blood Lipids and Cranial Nerve Involvement

#### 3.2.1. Comparison of General Characteristics Between Lipid Levels and Cranial Nerve Involvement

In this study, GBS patients with cranial nerve involvement (*n* = 112) were designated as the experimental group, while those without cranial nerve involvement (*n* = 70) served as the control group. The results revealed significant differences in the levels of LDL, ApoA, and ApoB), and Lp(a) between the two groups. Specifically, patients with cranial nerve involvement had significantly higher LDL levels compared with the control group, while their levels of ApoA, ApoB, and Lp(a) were significantly lower (Table 4).

#### 3.2.2. Logistic Regression Analysis of Lipid Levels and Cranial Nerve Involvement

Univariate logistic regression analysis revealed that after adjusting for potential confounding factors, elevated levels of LDL and ApoB in the lipid profile significantly increased the risk of cranial nerve involvement, while elevated levels of ApoA and the ApoA/B ratio exhibited protective effects (all *p* < 0.05) (Table 5).

**Figure 2:**
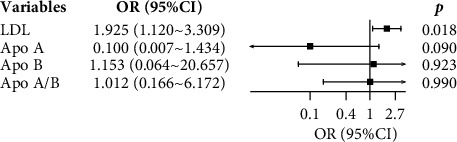
Forest plot of multivariate-adjusted associations between lipid profiles and cranial nerve involvement in Guillain–Barré syndrome.

After adjusting for potential risk factors, the multivariate logistic regression analysis model demonstrated that LDL remained a significant risk factor for predicting cranial nerve involvement (Figure 2).

**Figure 3:**
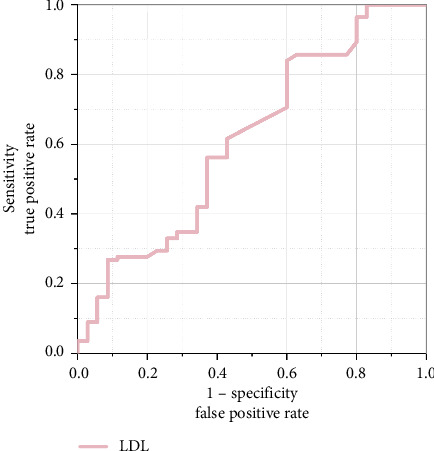
ROC curve analysis of LDL for predicting cranial nerve involvement. The area under the curve (AUC) was 0.612 (95% CI: 0.527–0.698, *p*=0.011), with an optimal cutoff value of 2.355 mmol/L demonstrating 83.9% sensitivity and 40.0% specificity.

Using the ROC curve with cranial nerve involvement as the outcome measure, we analyzed the diagnostic efficacy of LDL. The results showed AUC of 0.612 (95% CI: 0.527–0.698), *p*=0.011 (< 0.05), with an optimal cutoff value of 2.355 mmol/L. The sensitivity was 83.9% and specificity was 40.0%, suggesting that LDL levels ≥ 2.355 mmol/L may indicate symptomatic involvement of cranial nerves (Figure 3).

## 4. Discussion

GBS is an autoimmune-mediated peripheral neuropathy, typically triggered by infections, characterized by rapid progression of motor and sensory nerve dysfunction with potential life-threatening complications. Early identification of serological biomarkers for disease progression and severity assessment is critical for timely intervention and prognostic evaluation. In murine GBS models, elevated T-cell and macrophage levels drive immune activation and complement-mediated autoimmunity [2]. While dyslipidemia has been implicated in neuroinflammatory demyelinating disorders [8], its role in GBS remains underexplored [8]. Notably, reduced LDL and ApoB/ApoA1 ratios have been observed in GBS patients [1]. This retrospective study first systematically characterizes acute-phase lipid metabolic profiles in GBS, revealing that elevated LDL levels correlate with higher HFGS (indicative of disease severity) and cranial nerve involvement (AUC = 0.612, *p*=0.011), whereas increased ApoA levels associate with milder phenotypes. These findings underscore the potential mechanistic role of lipid dysregulation in GBS pathogenesis and progression [9].

This study conducted an in-depth analysis of the distribution characteristics and clinical significance of different types of cranial nerve involvement in mild and severe cases of GBS. The results showed that the proportion of oculomotor nerve involvement, facial nerve involvement, and involvement of lower cranial nerves (ninth and tenth pairs) was significantly higher in the severe group compared to the mild group (oculomotor nerve: *χ*^2^ = −7.11, *p* < 0.001; facial nerve: *χ*^2^ = −3.00, *p*=0.002; and lower cranial nerves: *χ*^2^ = −6.76, *p* < 0.001). Subtype analysis further revealed that oculomotor nerve involvement was particularly prominent in Miller Fisher Syndrome (MFS), which aligns with the pathological physiological characteristics of MFS, primarily affecting the brainstem and cerebellum. In contrast, facial nerve involvement was more common in the acute inflammatory demyelinating polyradiculoneuropathy (AIDP) subtype, which may reflect differences in immune attack patterns among subtypes. In addition, involvement of lower cranial nerves in severe cases (such as bulbar paralysis, often requiring mechanical ventilation support) underscores their importance in maintaining basic vital functions [10]. These findings suggest a close association between cranial nerve damage and disease severity, which may serve as a crucial indicator for prognosis assessment and clinical management.

This study utilized ROC curve analysis and found that an LDL level ≥ 2.215 mmol/L may indicate a more severe condition of GBS. This result is consistent with previous studies [5]. It has been discovered that high LDL levels can increase the number of lesions in multiple sclerosis, confirming a significant correlation between cholesterol metabolism pathways and multiple sclerosis [3]. In addition, low levels of LDL and ApoA are associated with poor prognosis and recurrence of autoimmune encephalitis, further supporting the potential link between lipid levels and neurological diseases [5]. High LDL levels may exacerbate the condition of GBS through the following mechanisms: (1) LDL and its oxidized form (oxLDL) can abnormally activate the immune system, inducing immune cell infiltration and cytokine release [11] and (2) their damaging effect on the myelin sheath structure may further intensify the inflammatory response and worsen clinical manifestations [12, 13], which aligns with the finding in this study that high LDL levels are associated with cranial nerve involvement. However, some studies have also pointed out [14] that a decrease in LDL levels during the acute phase may be related to changes in liver metabolism, suggesting that dynamic changes in LDL may have stage-specific characteristics.

Furthermore, ROC curve analysis revealed that an ApoA level ≤ 1.071 mg/dL may indicate a milder disease condition, suggesting that changes in ApoA levels could be closely related to lipid metabolism disorders in the pathological process [15]. The reduction in ApoA levels may be associated with the weakening of its anti-inflammatory and immunomodulatory functions [16]. As the main component of HDL, ApoA possesses anti-inflammatory and antioxidant properties. It not only promotes reverse cholesterol transport but also inhibits the release of proinflammatory factors (such as TNF-α, IL-1, and IL-6) by binding to immune cell receptors, thereby alleviating inflammatory responses [17]. In addition, ApoA may regulate T-cell function to promote immune tolerance [17], reducing immune attacks on neural tissues. At the same time, the neuroprotective effects of ApoA help mitigate neural damage and facilitate functional recovery.

The study results indicate that there is a correlation between the levels of LDL and ApoA upon hospital admission and the severity of GBS. Multivariate logistic regression analysis further confirmed that these factors are independent risk factors. Among the severe GBS patients included in this study, the average age was higher, and the prevalence of hypertension and diabetes was significantly higher compared with mild cases. Research has demonstrated that advanced age, hypertension, and diabetes are important risk factors for dyslipidemia, which may be closely related to systemic inflammation and lipid metabolism disorders caused by metabolic-related comorbidities. It is, therefore, hypothesized that advanced age and metabolic-related comorbidities (such as hypertension and diabetes) may increase the risk of dyslipidemia, consequently affecting the progression and severity of GBS. Although the multivariate model showed a statistically significant association between some lipid indicators and GBS severity, the AUC was only 0.69 and 0.62, suggesting that its predictive ability is at a medium-low level. This limitation indicates the potential interference of other unaccounted confounding factors.

In GBS patients with cranial nerve involvement, LDL levels were significantly elevated, while levels of ApoA, ApoB, and Lp(a) were significantly reduced. This further highlights the critical role of lipid metabolism disorders in the pathophysiology of GBS. Previous studies have confirmed that lipid abnormalities are associated with the development and progression of neuropathy [4], which is consistent with the findings of this study. The release of inflammatory factors during the acute phase may promote LDL synthesis by modulating liver metabolism while simultaneously inhibiting the production of HDL-related apolipoproteins, leading to lipid metabolism imbalance [17]. In a multifactorial adjusted model, LDL independently predicted the risk of cranial nerve involvement. Notably, the significance of ApoB in univariate analysis disappeared in the multivariate model, suggesting that the effect of LDL has a unique pathophysiological basis rather than merely reflecting the total number of atherogenic particles. The potential mechanisms include (1) elevated LDL, particularly oxLDL, can be phagocytosed by macrophages, leading to atherosclerosis in blood vessels supplying nerves, reducing blood flow, and impairing normal cranial nerve function [4]; (2) oxLDL generates excessive reactive oxygen species, attacking nerve cells and damaging cellular structures such as mitochondria and proteins, resulting in neuronal dysfunction; (3) oxLDL promotes the release of inflammatory factors, further exacerbating neural damage [6]; (4) high LDL levels may disrupt the blood–brain barrier, allowing harmful substances to enter the central nervous system, directly damaging cranial nerves, and disrupting the homeostasis of the neural microenvironment.

In summary, the findings of this study indicate that LDL and ApoA can serve as potential early biomarkers for assessing the severity of GBS and cranial nerve involvement, providing significant reference value for clinical diagnosis, timely intervention, and early prevention. The innovation of this study lies in being the first to explore the role of lipid levels and metabolism in GBS, offering new insights into evaluating disease severity and cranial nerve involvement. The results have practical clinical utility and can inform the development of individualized treatment strategies. In addition, this study comprehensively analyzed multiple potential influencing factors, further enriching the understanding of lipid metabolism indicators in GBS research and demonstrating considerable reference value and clinical application potential. The relationship between lipid metabolism and GBS, as well as other demyelinating diseases, may be influenced by various mechanisms, including metabolic regulation in inflammatory environments, immune modulation, and oxidative stress. Future research should further investigate the specific roles of these mechanisms and their dynamic changes during disease progression. Limitations of this study: (1) electrophysiological variations were not included in the analysis. Existing research has shown that electrophysiological variations are closely related to the extent of cranial nerve involvement in GBS patients and are particularly relevant in elderly individuals. Future studies could incorporate such data to comprehensively assess their clinical significance. (2) The study did not dynamically monitor the relationship between lipid levels and disease progression. Only the lipid levels upon hospital admission were analyzed, without accounting for changes during the recovery phase. (3) Patients' baseline metabolic status, such as a history of dyslipidemia, may have influenced the study results. (4) This was a single-center study, which may introduce center effects and has a relatively limited sample size, potentially affecting the generalizability and representativeness of the findings. Although multivariate logistic regression models suggested that certain lipid indicators are correlated with the severity of GBS, the AUC values were only 0.69 and 0.62, indicating that the predictive ability was only moderate. This suggests that the results should be interpreted in conjunction with other factors. Future studies should involve larger, multicenter, prospective research, particularly focusing on dynamically assessing changes in lipid metabolism indicators at different stages and their correlation with the pathogenesis, severity, and treatment outcomes of GBS to further validate and deepen the findings of this study. All authors have read and approved the final version of the manuscript. The corresponding author had full access to all of the data in this study and takes complete responsibility for the integrity of the data and the accuracy of the data analysis.

## Figures and Tables

**Table 1:** Comparison of general information between the severe and mildly ill groups.

Variables	Mild group (*n* = 70)	Severe group (*n* = 112)	*χ* ^2^/*Z*/*t*	*p*
Age^#^	50.00 (44.00, 57.00)	60.00 (52.25, 66.00)	−4.58	< 0.001^∗^
Sex (male)^≠^	54 (77.14)	96 (85.71)	2.18	0.139
Sensory impairment^≠^	66 (94.29)	112 (100.00)	4.16	0.042^∗^
Motor disorders^≠^	66 (94.29)	112 (100.00)	4.16	0.042^∗^
Oculomotor nerve involvement, *n* (%)^≠^	16 (22.86)	86 (76.79)	−7.11	< 0.001^∗^
Inferior cranial nerve involvement, *n* (%)^≠^	4 (5.71)	62 (55.36)	−6.76	< 0.001^∗^
Diabetes^≠^	15 (21.43)	44 (39.64)	6.48	0.011^∗^
Hypertension^≠^	7 (10.00)	37 (33.04)	12.47	< 0.001^∗^
Total protein g/L^∆^	67.52 ± 8.52	69.25 ± 9.74	−1.22	0.223
ALT^#^	20.00 (14.88, 28.32)	21.25 (16.00, 31.30)	−0.83	0.406
AST^#^	19.90 (17.43, 29.32)	22.10 (16.52, 29.25)	−0.66	0.510
GGT^#^	24.00 (17.10, 37.60)	29.00 (22.00, 44.25)	−2.16	0.031^∗^
Total bilirubin^#^	11.30 (8.10, 13.93)	10.40 (6.68, 14.48)	−0.44	0.661
Direct bilirubin^#^	2.39 (1.45, 3.55)	1.90 (0.03, 3.50)	−1.43	0.152
Indirect bilirubin^#^	7.69 (5.74, 9.75)	6.50 (4.44, 8.92)	−1.78	0.075
Alkaline phosphatase^#^	70.00 (60.10, 89.00)	84.71 (63.75, 104.25)	−2.01	0.045^∗^
Creatinine^#^	57.80 (45.45, 68.57)	55.65 (48.00, 71.15)	−0.76	0.446
Uric acid^#^	285.84 (225.38, 333.23)	295.00 (219.47, 357.50)	−0.07	0.941
Total cholesterol^#^	4.49 (3.93, 5.12)	4.20 (3.48, 4.70)	−2.40	0.016^∗^
Triglycerides^#^	1.75 (0.90, 2.12)	1.35 (0.91, 1.79)	−1.34	0.182
LDL^#^	2.60 (2.30, 3.09)	2.83 (2.40, 3.53)	−2.25	0.024^∗^
HDL^#^	1.11 (1.04, 1.27)	1.10 (0.85, 1.28)	−2.36	0.019^∗^
ApoA^#^	1.11 (1.05, 1.24)	1.05 (0.97, 1.05)	−4.45	< 0.001^∗^
ApoB^#^	0.96 (0.84, 0.99)	0.96 (0.94, 0.96)	−1.20	0.231
Apo A/B^#^	1.22 (1.10, 1.48)	1.22 (1.10, 1.22)	−2.14	0.032^∗^
Lipoprotein^#^	276.08 (116.00, 283.52)	276.08 (216.25, 276.08)	−1.49	0.135

^∗^Represents a *p* value of less than 0.05.

^#^Represents non-normally distributed data confirmed by the SK normality test.

^∆^Represents having passed the SK normality test.

^≠^Indicates rank information.

**Table 2:** Univariate logistic regression analysis.

Variables	*β*	S.E	*Z*	*p*	OR (95% CI)
Diabetes	0.88	0.35	2.51	0.012^∗^	2.41 (1.21–4.78)
Hypertension	1.49	0.45	3.34	< 0.001^∗^	4.44 (1.85–10.65)
Total protein	0.02	0.02	1.22	0.223	1.02 (0.99–1.05)
ALT	−0.00	0.01	−0.13	0.895	1.00 (0.99–1.01)
AST	0.01	0.01	0.93	0.353	1.01 (0.99–1.04)
GGT	−0.00	0.00	−0.32	0.745	1.00 (0.99–1.01)
Total bilirubin	0.01	0.03	0.34	0.737	1.01 (0.96–1.06)
Direct bilirubin	−0.09	0.08	−1.18	0.237	0.91 (0.78–1.06)
Indirect bilirubin	−0.02	0.03	−0.57	0.571	0.98 (0.92–1.05)
Alkaline phosphatase	0.01	0.01	1.82	0.069	1.01 (1.00–1.02)
Creatinine	0.01	0.01	1.31	0.192	1.01 (1.00–1.02)
Total cholesterol	−0.13	0.12	−1.06	0.291	0.88 (0.69–1.12)
Triglycerides	−0.12	0.11	−1.10	0.272	0.89 (0.72–1.10)
LDL	0.70	0.24	2.91	0.004^∗^	2.02 (1.26–3.25)
HDL	−1.23	0.63	−1.94	0.053	0.29 (0.08–1.01)
ApoA	−3.12	0.96	−3.26	0.001^∗^	0.04 (0.01–0.29)
ApoB	0.70	0.67	1.04	0.299	2.01 (0.54–7.51)
ApoA/B	−0.81	0.38	−2.13	0.034^∗^	0.45 (0.21–0.94)
Lipoprotein A	0.00	0.00	0.32	0.748	1.00 (1.00–1.00)

^∗^Represents a *p* value of less than 0.05.

**Table 3:** Multivariate logistic regression analysis.

Variables	*β*	S.E	Z	*p*	OR (95%CI)
LDL	0.918	0.351	6.849	0.009^∗^	2.503 (1.259–4.977)
ApoA	−5.486	0.515	14.112	< 0.001^∗^	0.004 (0.001–0.073)
ApoA/B	0.817	0.630	1.679	0.195	2.263 (0.658–7.781)
Diabetes	0.567	0.416	1.855	0.173	1.762 (0.780–3.982)
Hypertension	1.419	0.515	7.604	0.006^∗^	4.134 (1.508–11.337)

^∗^Represents a *p* value of less than 0.05.

**Table 4:** Comparison of lipid levels between patients with and without cranial nerve involvement.

	Experimental group (*n* = 112)	Control group (*n* = 70)	*Z*	*p*
Total cholesterol^#^	4.29 (3.67–5.01)	4.50 (3.56–5.12)	−0.637	0.524
Triglycerides^#^	1.39 (0.86–1.79)	1.48 (0.95–2.12)	−1.547	0.115
LDL^#^	2.83 (2.43–3.53)	2.60 (2.24–3.38)	−2.547	0.011^∗^
HDL^#^	1.10 (0.86–1.27)	1.11 (1.01–1.28)	−1.338	0.181
ApoA^#^	1.05 (0.98–1.05)	1.11 (1.05–1.23)	−4.073	< 0.001^∗^
ApoB^#^	0.96 (0.96–0.96)	0.96 (0.72–0.98)	−2.366	0.018^∗^
ApoA/B^#^	1.22 (1.02–1.22)	1.22 (1.10–1.50)	−2.211	0.027^∗^
Lipoprotein(a)^#^	218.08 (119.00–276.08)	276.08 (119.00–276.08)	−2.465	0.014^∗^

^∗^Represents a *p* value of less than 0.05.

^#^Represents non-normally distributed data confirmed by the SK normality test.

**Table 5:** Univariate logistic regression analysis.

Variables	*β*	S.E	Z	*p*	OR (95% CI)
Total cholesterol	−0.111	0.123	−0.901	0.367	0.895 (0.703–1.139)
Triglycerides	−0.078	0.108	−0.721	0.471	0.925 (0.748–1.143)
HDL	−0.645	0.622	−1.036	0.300	0.525 (0.155–1.778)
LDL	0.614	0.237	2.595	0.009^∗^	1.849 (1.162–2.940)
ApoA^#^	−2.084	0.877	−2.378	0.017^∗^	0.124 (0.022–0.694)
ApoB^#^	1.423	0.712	1.999	0.046^∗^	4.148 (1.028–16.734)
ApoA/B^#^	−0.962	0.395	−2.435	0.015^∗^	0.382 (0.176–0.829)
LDL ApoB	0.080	0.175	0.460	0.645	1.084 (0.769–1.526)
HDL ApoA	0.366	0.479	0.764	0.445	1.442 (0.564–3.690)
Lipoprotein(a)	0.002	0.001	1.768	0.077	1.002 (1.000–1.004)

^∗^Represents a *p* value of less than 0.05.

## Data Availability

The corresponding author, Juan Tang, affirms that this manuscript is an honest, accurate, and transparent account of the study being reported; that no important aspects of the study have been omitted; and that any discrepancies from the study as planned (and, if relevant, registered) have been explained. The data supporting the findings of this study are available from the corresponding author upon reasonable request.
